# The role of sexual preferences in intrasexual female competition

**DOI:** 10.1186/1471-2148-12-218

**Published:** 2012-11-14

**Authors:** Alicia M Frame

**Affiliations:** 1 Department of Biology, University of North Carolina, Chapel Hill, North Carolina, 27599, USA

**Keywords:** Sexual selection, Mate choice, Female preference, Competition, Population genetics

## Abstract

**Background:**

While search costs have long been understood to affect the evolution of female preference, other costs associated with mating have been the focus of much less attention. Here I consider a novel mate choice cost: female-female intrasexual competition, that is, when females compete with each other for mates. This competition results in cost to female fecundity, such as a reduction in fertility due to decreased direct benefits, sperm limitation, or time and resources spent competing for a mate. I asked if female-female competition affects the evolution of preferences, and further, if the presence of multiple, different, preferences in a population can reduce competitive costs.

**Results:**

Using population genetic models of preference and trait evolution, I found that intrasexual competition leads to direct selection against female preferences, and restricts the parameter space under which preference may evolve. I also examined how multiple, different, preferences affected preference evolution with female intrasexual competition.

**Conclusions:**

Multiple preferences primarily serve to increase competitive costs and decrease the range of parameters under which preferences may evolve.

## Background

Costs associated with female preferences are often assumed to be directly related to the act of searching for a preferred mate: 1) time spent searching for a mate, 2) the potential for a choosy female to go unmated, or 3) an increased risk of predation [1]. These previously considered costs are viability costs, where the female’s chances of survival and successful mating are affected; here I present an argument for the role of fertility costs and their effect on preference evolution. There is great potential for costs involved in mate choice to be derived from female-female intrasexual competition as well. In general, these costs have not been widely studied or taken into account as potential selective forces driving (or preventing) female preference evolution [2].

In resource-based polygyny, males provide females with resources such as parental care, defense, or territories in which to raise their young. In such scenarios, the cost of competing for a desired male is clear cut: it is well accepted that males may only support a limited number of females, and increasing beyond that threshold leads to decreased female reproductive fitness [3]. Even in systems where resource limitations are less obvious, reduction in parental efforts can lead to decreased female fitness. For example, in dendrobatid frogs, brood sizes decreased significantly after multiple matings due to decreased male parental effort [4]. Similarly, in polygynous tree swallows, females mated with polygynous males had reduced fitness because of decreased parental care [5].

Even in polygynous species where males offer little to females, females may still incur costs simply by waiting to mate with a preferred male, by competing with other females for a preferred male’s attention, or by suffering reduced fecundity from male sperm depletion. In lekking birds, dominant females monopolizing preferred males time can lead to delayed breeding and decreased reproductive fitness [6]. Females may also respond to competition for males with direct aggression, potentially injuring competitors [7,8]. Sperm depletion and exhaustion, due to males mating multiply, may be costly to females as well [9]. Sperm exhaustion has been tied to reduced reproductive fitness for females in insects [10], fish [11], and crustaceans [12-14]. Although these costs are small compared to those suffered by females mating in resource based polygyny, they are all associated with significant decreases in reproductive fitness.

In all of these situations, females are likely to experience a cost for preferring ‘popular’ males, i.e. those who have many mates. In fact, when females suffer fitness reductions from mating with sperm depleted males, if they can accurately assess the number of mates a male has, they choose males with fewer mates [12]. In general, however, it may be difficult for females to ascertain whether they are likely to suffer competitive costs: for example, in systems where males have large or overlapping territories, females have little or no information about additional mates; in systems where males provide resources that cannot easily be quantified, the female may have no information about these costs whatsoever.

Without direct knowledge, what can females do to avoid costly competition? One possibility is that multiple preferences may aid in alleviating or preventing competition. Indeed, many of the species discussed previously as examples of costly female competition have multiple male traits and preferences as well (guppies: [15]; tree swallows: [16]; Great Snipe: [17]). If females have differing preferences, and if males display differing traits, then competition could be reduced. For example, if females of some species may prefer complex song, long tails, or both, and males may have one or both of those traits; females choosing mates with high quality plumage may reduce their cost of competition because they are not competing with those who choose males with a complex song.

Empiricists have found cases of repeatable variability in genetically determined female preferences [18,19]. In such scenarios, females appear to be selecting mates based on multiple independent male traits. Marchetti [19] found evidence that female yellow browed leaf warblers based their choice of mate on several male characters, and although females preferred high quality males, different females used different traits to distinguish between these males [20]. Not only demonstrated multiple preferences in female guppies, but demonstrated that they are heritable and genetically independent. The genetic assumptions of my model are built upon these findings.

Although there is ample empirical evidence of intrasexual mate competition in females, to my knowledge it has not been incorporated into evolutionary models. Fawcett and Johnstone [21] considered the potential for female competition to alter mate choice from a game theoretic point of view, and showed that female competition could alter mating decisions. However, their model ignored genetics and focused primarily on alternative strategies, which is problematic because linkage disequilibrium between genes is a powerful evolutionary force. I chose to use a population genetic model which explicitly considers distinct genotypes and the potential for non-random association between loci (linkage disequilibrium) to evolve via assortative mating, leading to indirect selection on preference and traits.

Here, I argue that competition alone, regardless of the type of trait possessed by males, will impact preference evolution. To address these issues, I first model the evolution of a single female preference in a system with costly intrasexual competition for mates, to determine when preferences may still evolve and the strength of selection acting on preference. Then, I consider whether or not the presence of an additional female preference alleviates competitive costs, and how selection on preferences changes with the introduction of an additional preference. When discussing multiple preferences, I am referring to multiple preferences controlled by independent loci: females may have no preferences, a single preference, or both. As novel preferences evolve to fixation, the result is that the majority of females possess both preferences.

## Model specification and results

I model mate choice with costly female competition for mates using a population genetic model with haploid loci and discrete non-overlapping generations, based on previous models of sexual selection via female choice [22]. The model assumes polygyny; all females mate, but males have variable mating success.

For each model, I begin by describing the life cycle in terms of birth, mating, fertility selection, and zygote formation. Using these equations, I can then calculate the strength of direct selection on preference using the notation of Barton and Turelli [23].

### One preference, one trait (two locus model)

Female preference and male traits are controlled by two haploid loci, each with two alleles: the preferences locus, P, controls female preference, and the trait locus, T, controls male traits. Uppercase letters indicate the presence of a preference or trait, lowercase letters indicate the absence. These two loci yield four genotypes: PT, Pt, pT, and pt. I denote their frequencies as x_1_, *x*_2_, x_3_, and x_4_; X_T_ is used to denote the frequency of the male trait allele (x_1_ + x_3_), and X_P_ is used to denote the frequency of the female preference allele (x_1_ + *x*_2_).

Females choose mates based on their preferences. A female without the preference allele (a p female) will mate randomly with respect to male genotype, whereas a female with the preference allele (a P female) is *α* times more likely to mate with a male possessing the trait allele, given that she has evaluated one of each type.

Mate choice results in a 4x4 matrix, F, whose elements F_ij_ represent the proportion of matings taking place between genotypes i and j:

(1) $$F_{\mathrm{ij}}=\frac{k_{\mathrm{ij}}\alpha \cdot x_{i}x_{j}}{Z_{i}}\text{,}$$

Where k_ij_ is the modifier of preference strength for an x_i_ female mating with an x_j_ male; k_11_ and k_13_ are 1 (x_1_ and *x*_2_ females prefer x_1_ and x_3_ males), all others are 0 (x_3_ and x_4_ females mate randomly; x_1_ and *x*_2_ females do not prefer *x*_2_ and x_4_ (traitless) males). Z_i_ is a normalization to ensure that all female genotypes have equal mating success; *Z*_1_ = *α X*_*T*_ + (1 − *X*_*T*_), *Z*_2_ = 1. The full mating table is given in Table 1.

**Table 1:** Mating table for one preference/one trait model

		**Males**			
		**x1**	**x2**	**x3**	**x4**
Females	x1	$\frac{\text{α}\left({\text{x}}_{1}{\text{x}}_{1}\right)}{Z}$	$\frac{\left({\text{x}}_{1}{\text{x}}_{2}\right)}{Z}$	$\frac{\text{α}\left({\text{x}}_{1}\text{x}3\right)}{Z}$	$\frac{\left({\text{x}}_{1}{\text{x}}_{4}\right)}{Z}$
	x2	$\frac{\text{α}\left({\text{x}}_{2}{\text{x}}_{1}\right)}{Z}$	$\frac{\left({\text{x}}_{2}{\text{x}}_{2}\right)}{Z}$	$\frac{\text{α}\left({\text{x}}_{2}{\text{x}}_{3}\right)}{Z}$	$\frac{\left({\text{x}}_{2}{\text{x}}_{4}\right)}{Z}$
	x3	x_3_x_1_	x_3_x_2_	x_3_x_3_	x_3_x_4_
	x4	x_4_x_1_	x_4_x_2_	x_4_x_3_	x_4_x_4_
	Z = α(x_1_ + x_3_) + x_2_ + x_4_				

Females with the preference allele prefer males bearing a trait by a factor α. Matings are normalized by Z so that all female genotypes have equal mating success.

After females have selected mates, fertility selection is exerted against the offspring of males with a surplus of mates. I denote the intensity of fertility selection by γ. When the mating frequency of a particular male genotype exceeds the population frequency of that male genotype, fertility is reduced proportionally. For genotype *j*, fertility selection is determined by

(2) $$\phi_{j}=\gamma \left(\frac{\sum_{i=1}^{4}F_{i,j}}{x_{j}}-1\right)\text{.}$$

*F*^*φ*^, the fertility selection matrix, is calculated by multiplying each column of F by the corresponding fertility reduction suffered by the male parental genotype. The result is that

(3) $$F_{\mathrm{ij}}^{\phi }=F_{\mathrm{ij}}\left(1-\phi_{j}\right)\text{.}$$

Recombination follows sexual selection and fertility selection; recombination rates are assumed to be 0.5 between all loci for simplicity (free recombination).

**Figure 1:**
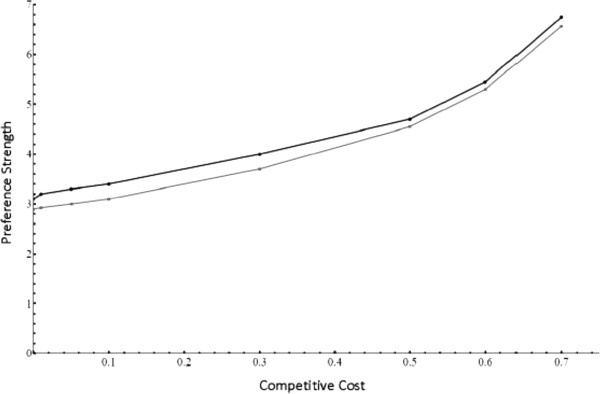
**Preference strength necessary for preference evolution.** This figure shows the minimum preference strength necessary for a female preference allele to evolve to fixation. The x axis shows the competitive cost (the value of γ) for a given simulation, and the y axis shows the necessary preference strength to be able to evolve female preferences (*α*). As the competitive cost increases, higher preference strengths are necessary to fix female preferences. The black line is the preference strength needed for simulations with a single preference, the grey line is the preference strength required in simulations with two preferences (both introduced at low initial frequencies).

Using these life cycle equations, I first used numerical simulations (run in Matlab) to confirm that it was possible to evolve preferences despite competitive costs. Female preferences may still evolve with competitive costs, although the preference strength needed to overcome selection and fix preferences increases as costs become greater (Figure 1, black line). This confirms that 1) female-female competition does act as a previously unexamined cost of choice, making it likely to cause natural selection against preference evolution, and 2) this cost does not completely bar preference evolution.

To measure how strong natural selection against costly female preferences is, I applied the methodology of Barton and Turelli [23] to calculate the strength of direct selection of preference. To illustrate the role of direct and indirect selection, one can write a general equation for the change in the frequency of preference alleles between generations:

(4) $$\Delta p=a_{P,0}C_{\mathrm{PP}}+a_{T,0}C_{\mathrm{PT}}$$

Here, Δp is the sum of direct selection and indirect selection. For any two loci X and Y, *a*_*X,*0_*C*_*XY*_ measures how the frequency of an allele at locus Y changes due to the selection at locus X *(a*_*X,*0_*)* and the genetic association between locus X and Y (*C*_*XY*_). Thus, change in preference is driven by direct selection on preferences, *a*_*P,*0_*C*_*PP*_, as well as indirect selection via the linkage disequilibrium between preference and trait, *a*_*T,*0_*C*_*PT*_ (from [23], eq 16).

Equation (4) can then be used to partition out how much change in the frequency of a preference allele is due to direct versus indirect selection. The first term represents change due to direct selection:

(5) $$\text{Δ}p_{\text{direct}}=a_{P,0}C_{\mathrm{PP}}\text{.}$$

This represents direct selection on locus P_i_, favoring preference, with strength *a*_*(P,*0*)*_, multiplied by the genetic variance at the P_i_ locus, *C*_*PP*_.

The procedure for solving for direct selection, *a*_*P,*0_, is described in Appendix 1. The result is that we have an equation describing the strength of selection for (or against) possessing a preference allele:

(6) $$a_{P,0}=-\frac{{\left(\alpha -1\right)}^{2}\left(P-1\right)\left(\left(1-T\right)+D_{P,T}\left(1-2T\right)\right)\cdot \gamma }{{\left(\alpha +T-\alpha T\right)}^{2}}$$

Where *P* is the frequency of the preference allele, *T* is the frequency of the trait allele, and *D*_*P,T*_ is the linkage disequilibrium between preference and trait.

In terms of selective forces, equation (6) demonstrates the selection on the preference locus is a function of preference and trait frequency, as well as preference strength and the cost of competition – all of which is intuitive from the model description. To understand what (6) means in more concrete terms, I first proved that the expression is always negative for realistic values of *P* and *T* (*P,T*≤1). The sign of (6) is negative when (1 − *T*) + *D*_*P*,*T*_(1 − 2*T*) > 0. Thus,

$$1-T+D_{P,T}-2D_{P,T}T>0$$

$$1+D_{P,T}>T\left(1+2D_{P,T}\right)$$

(7) $$\frac{1+D_{P,T}}{1-2D_{P,T}}>T$$

Because 1≥*T*≥0, and linkage between preference and trait is greater than or equal to 0, the right hand side of (7) is always positive, and, in turn, (6) is always negative.

**Figure 2:**
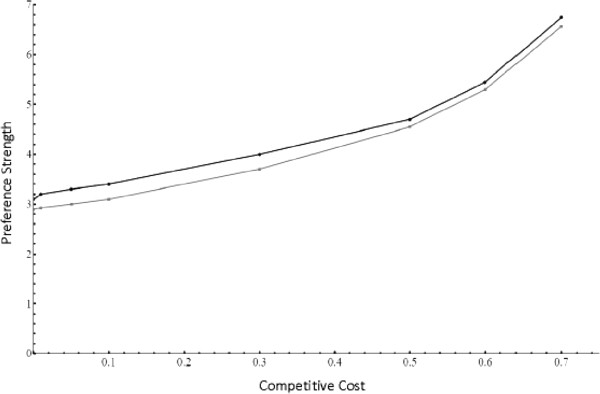
**Direct selection for a single preference.** This figure shows the strength of direct selection against female preference (*ã*_*p*,0_) as male trait frequency increases from 0 – 1. Each line represents direct selection for a different combination of female preference frequency (P) and cost (γ) – the solid black line indicates P=.01, γ=.01; solid gray indicates P=.01, γ=.1; dashed black indicates P=.5, γ=.01; dashed grey indicates P=.5, γ=.1. For all simulations, *α*=5; changing preference strength did not change the shape of the cost curve, but only scaled up the strength of selection against preference. Direct selection always acts against preferences, except when male traits are absent or fixed, in which case, *a*_*p*,0_=0.

I plotted *a*_*P*,0_ for varying frequencies of preference and trait alleles, as well as different cost regimes (Figure 2). Because the value of *α* does not change the shape of the curve, I only display results with *α* = 5. For all scenarios with female competition for preferred males, *a*_*P*,0_ is negative (if γ=0 or *α*=1, *ã*_*P*,0_=0). This means that direct selection always acts against female preference if competition is a factor; male traits in this scenario will only evolve if female preferences are sufficiently strong such that indirect (sexual) selection can outweigh direct selection.

### Two preferences, two traits (four locus model)

Having shown that a single preference is selected against when females compete, I now consider whether or not a second preference is sufficient to alleviate competition, leading to direct selection for preferences.

In this model, there are an additional two loci: two preference loci, P_1_ and P_2_, control female preference, and two trait loci, T_1_ and T_2_, control male display traits. These four loci yield 2^4^= 16 genotypes: P_1_P_2_T_1_T_2_, P_1_P_2_T_1_t_2_, P_1_P_2_t_1_T_2_, P_1_P_2_t_1_t_2_, P_1_p_2_T_1_T_2_, P_1_p_2_T_1_t_2_, and so on. I denote their frequencies by *x*_1_, *x*_2_, …, *x*_16_.

As before, females select mates based on their preferences. P_1_ corresponds to a preference for trait one and P_2_ corresponds to a preference for trait 2. For example, a P_1_p_2_ female prefers males possessing the T_1_ trait. When a female possesses both preference alleles, P_1_P_2_, she is *α* times as likely to mate with a T_1_t_2_ or t_1_T_2_ male, and 1.5 *α* times as likely to mate with a T_1_T_2_ male, assuming she has encountered one of each type of male. Mate choice and fertility selection are calculated as described in equations (1), (2) and (3). The full mating table is given in Table 2.

**Table 2:** Mating table for two preference/two trait model

		**Males**															
**Females**		**x**_**1**_	**x**_**2**_	**x**_**3**_	**x**_**4**_	**x**_**5**_	**x**_**6**_	**x**_**7**_	**x**_**8**_	**x**_**9**_	**x**_**10**_	**x**_**11**_	**x**_**12**_	**x**_**13**_	**x**_**14**_	**x**_**15**_	**x**_**16**_
	**x**_**1**_	$\frac{\alpha p\;\left(x_{1}x_{1}\right)}{Z_{1}}$	$\frac{\alpha \;\left(x_{1}x_{2}\right)}{Z_{1}}$	$\frac{\alpha \;\left(x_{1}x_{3}\right)}{Z_{1}}$	$\frac{\;\left(x_{1}x_{4}\right)}{Z_{1}}$	$\frac{\alpha p\;\left(x_{1}x_{5}\right)}{Z_{1}}$	$\frac{\alpha \;\left(x_{1}x_{6}\right)}{Z_{1}}$	$\frac{\alpha \;\left(x_{1}x_{7}\right)}{Z_{1}}$	$\frac{\;\left(x_{1}x_{8}\right)}{Z_{1}}$	$\frac{\alpha p\;\left(x_{1}x_{9}\right)}{Z_{1}}$	$\frac{\alpha \;\left(x_{1}x_{10}\right)}{Z_{1}}$	$\frac{\alpha \;\left(x_{1}x_{11}\right)}{Z_{1}}$	$\frac{\;\left(x_{1}x_{12}\right)}{Z_{1}}$	$\frac{\alpha p\;\left(x_{1}x_{13}\right)}{Z_{1}}$	$\frac{\alpha \;\left(x_{1}x_{14}\right)}{Z_{1}}$	$\frac{\alpha \;\left(x_{1}x_{15}\right)}{Z_{1}}$	$\frac{\;\left(x_{1}x_{16}\right)}{Z_{1}}$
	X_2_	$\frac{\alpha p\;\left(x_{2}x_{1}\right)}{Z_{1}}$	$\frac{\alpha \;\left(x_{2}x_{2}\right)}{Z_{1}}$	$\frac{\alpha \;\left(x_{2}x_{3}\right)}{Z_{1}}$	$\frac{\;\left(x_{2}x_{4}\right)}{Z_{1}}$	$\frac{\alpha p\;\left(x_{2}x_{5}\right)}{Z_{1}}$	$\frac{\alpha \;\left(x_{2}x_{6}\right)}{Z_{1}}$	$\frac{\alpha \;\left(x_{2}x_{7}\right)}{Z_{1}}$	$\frac{\;\left(x_{2}x_{8}\right)}{Z_{1}}$	$\frac{\alpha p\;\left(x_{2}x_{9}\right)}{Z_{1}}$	$\frac{\alpha \;\left(x_{2}x_{10}\right)}{Z_{1}}$	$\frac{\alpha \;\left(x_{2}x_{11}\right)}{Z_{1}}$	$\frac{\;\left(x_{2}x_{12}\right)}{Z_{1}}$	$\frac{\alpha p\;\left(x_{2}x_{13}\right)}{Z_{1}}$	$\frac{\alpha \;\left(x_{2}x_{14}\right)}{Z_{1}}$	$\frac{\alpha \;\left(x_{2}x_{15}\right)}{Z_{1}}$	$\frac{\;\left(x_{2}x_{16}\right)}{Z_{1}}$
	X_3_	$\frac{\alpha p\;\left(x_{3}x_{1}\right)}{Z_{1}}$	$\frac{\alpha \;\left(x_{3}x_{2}\right)}{Z_{1}}$	$\frac{\alpha \;\left(x_{3}x_{3}\right)}{Z_{1}}$	$\frac{\;\left(x_{3}x_{4}\right)}{Z_{1}}$	$\frac{\alpha p\;\left(x_{3}x_{5}\right)}{Z_{1}}$	$\frac{\alpha \;\left(x_{3}x_{6}\right)}{Z_{1}}$	$\frac{\alpha \;\left(x_{3}x_{7}\right)}{Z_{1}}$	$\frac{\;\left(x_{3}x_{8}\right)}{Z_{1}}$	$\frac{\alpha p\;\left(x_{3}x_{9}\right)}{Z_{1}}$	$\frac{\alpha \;\left(x_{3}x_{10}\right)}{Z_{1}}$	$\frac{\alpha \;\left(x_{3}x_{11}\right)}{Z_{1}}$	$\frac{\;\left(x_{3}x_{12}\right)}{Z_{1}}$	$\frac{\alpha p\;\left(x_{3}x_{13}\right)}{Z_{1}}$	$\frac{\alpha \;\left(x_{3}x_{14}\right)}{Z_{1}}$	$\frac{\alpha \;\left(x_{3}x_{15}\right)}{Z_{1}}$	$\frac{\;\left(x_{3}x_{16}\right)}{Z_{1}}$
	X_4_	$\frac{\alpha p\;\left(x_{4}x_{1}\right)}{Z_{4}}$	$\frac{\alpha \;\left(x_{4}x_{2}\right)}{Z_{1}}$	$\frac{\alpha \;\left(x_{4}x_{3}\right)}{Z_{1}}$	$\frac{\;\left(x_{4}x_{4}\right)}{Z_{1}}$	$\frac{\alpha p\;\left(x_{4}x_{5}\right)}{Z_{1}}$	$\frac{\alpha \;\left(x_{4}x_{6}\right)}{Z_{1}}$	$\frac{\alpha \;\left(x_{4}x_{7}\right)}{Z_{1}}$	$\frac{\;\left(x_{4}x_{8}\right)}{Z_{1}}$	$\frac{\alpha p\;\left(x_{4}x_{9}\right)}{Z_{1}}$	$\frac{\alpha \;\left(x_{4}x_{10}\right)}{Z_{1}}$	$\frac{\alpha \;\left(x_{4}x_{11}\right)}{Z_{1}}$	$\frac{\;\left(x_{4}x_{12}\right)}{Z_{1}}$	$\frac{\alpha p\;\left(x_{4}x_{13}\right)}{Z_{1}}$	$\frac{\alpha \;\left(x_{4}x_{14}\right)}{Z_{1}}$	$\frac{\alpha \;\left(x_{4}x_{15}\right)}{Z_{1}}$	$\frac{\;\left(x_{1}x_{16}\right)}{Z_{1}}$
	x_5_	$\frac{\alpha \;\left(x_{5}x_{2}\right)}{Z_{2}}$	$\frac{\alpha \;\left(x_{5}x_{2}\right)}{Z_{2}}$	$\frac{\;\left(x_{5}x_{3}\right)}{Z_{2}}$	$\frac{\;\left(x_{5}x_{4}\right)}{Z_{2}}$	$\frac{\alpha \;\left(x_{5}x_{5}\right)}{Z_{2}}$	$\frac{\alpha \;\left(x_{5}x_{6}\right)}{Z_{2}}$	$\frac{\left(x_{5}x_{7}\right)}{Z_{2}}$	$\frac{\;\left(x_{5}x_{8}\right)}{Z_{2}}$	$\frac{\alpha \left(x_{5}x_{9}\right)}{Z_{2}}$	$\frac{\alpha \;\left(x_{5}x_{10}\right)}{Z_{2}}$	$\frac{\;\left(x_{5}x_{11}\right)}{Z_{2}}$	$\frac{\;\left(x_{5}x_{12}\right)}{Z_{2}}$	$\frac{\alpha \;\left(x_{5}x_{13}\right)}{Z_{2}}$	$\frac{\alpha \;\left(x_{5}x_{14}\right)}{Z_{2}}$	$\frac{\;\left(x_{5}x_{15}\right)}{Z_{2}}$	$\frac{\;\left(x_{5}x_{16}\right)}{Z_{2}}$
	x_6_	$\frac{\alpha \;\left(x_{6}x_{2}\right)}{Z_{2}}$	$\frac{\alpha \;\left(x_{6}x_{2}\right)}{Z_{2}}$	$\frac{\;\left(x_{6}x_{3}\right)}{Z_{2}}$	$\frac{\;\left(x_{6}x_{4}\right)}{Z_{2}}$	$\frac{\alpha \;\left(x_{6}x_{5}\right)}{Z_{2}}$	$\frac{\alpha \;\left(x_{6}x_{6}\right)}{Z_{2}}$	$\frac{\left(x_{6}x_{7}\right)}{Z_{2}}$	$\frac{\;\left(x_{6}x_{8}\right)}{Z_{2}}$	$\frac{\alpha \left(x_{6}x_{9}\right)}{Z_{2}}$	$\frac{\alpha \;\left(x_{6}x_{10}\right)}{Z_{2}}$	$\frac{\;\left(x_{6}x_{11}\right)}{Z_{2}}$	$\frac{\;\left(x_{6}x_{12}\right)}{Z_{2}}$	$\frac{\alpha \;\left(x_{6}x_{13}\right)}{Z_{2}}$	$\frac{\alpha \;\left(x_{6}x_{14}\right)}{Z_{2}}$	$\frac{\;\left(x_{6}x_{15}\right)}{Z_{2}}$	$\frac{\;\left(x_{6}x_{16}\right)}{Z_{2}}$
	x_7_	$\frac{\alpha \;\left(x_{7}x_{2}\right)}{Z_{2}}$	$\frac{\alpha \;\left(x_{7}x_{2}\right)}{Z_{2}}$	$\frac{\;\left(x_{7}x_{3}\right)}{Z_{2}}$	$\frac{\;\left(x_{7}x_{4}\right)}{Z_{2}}$	$\frac{\alpha \;\left(x_{7}x_{5}\right)}{Z_{2}}$	$\frac{\alpha \;\left(x_{7}x_{6}\right)}{Z_{2}}$	$\frac{\left(x_{7}x_{7}\right)}{Z_{2}}$	$\frac{\;\left(x_{7}x_{8}\right)}{Z_{2}}$	$\frac{\alpha \left(x_{7}x_{9}\right)}{Z_{2}}$	$\frac{\alpha \;\left(x_{7}x_{10}\right)}{Z_{2}}$	$\frac{\;\left(x_{7}x_{11}\right)}{Z_{2}}$	$\frac{\;\left(x_{7}x_{12}\right)}{Z_{2}}$	$\frac{\alpha \;\left(x_{7}x_{13}\right)}{Z_{2}}$	$\frac{\alpha \;\left(x_{7}x_{14}\right)}{Z_{2}}$	$\frac{\;\left(x_{7}x_{15}\right)}{Z_{2}}$	$\frac{\;\left(x_{7}x_{16}\right)}{Z_{2}}$
	x_8_	$\frac{\alpha \;\left(x_{8}x_{2}\right)}{Z_{2}}$	$\frac{\alpha \;\left(x_{8}x_{2}\right)}{Z_{2}}$	$\frac{\;\left(x_{8}x_{3}\right)}{Z_{2}}$	$\frac{\;\left(x_{8}x_{4}\right)}{Z_{2}}$	$\frac{\alpha \;\left(x_{8}x_{5}\right)}{Z_{2}}$	$\frac{\alpha \;\left(x_{8}x_{6}\right)}{Z_{2}}$	$\frac{\left(x_{8}x_{7}\right)}{Z_{2}}$	$\frac{\;\left(x_{8}x_{8}\right)}{Z_{2}}$	$\frac{\alpha \left(x_{8}x_{9}\right)}{Z_{2}}$	$\frac{\alpha \;\left(x_{8}x_{10}\right)}{Z_{2}}$	$\frac{\;\left(x_{8}x_{11}\right)}{Z_{2}}$	$\frac{\;\left(x_{8}x_{12}\right)}{Z_{2}}$	$\frac{\alpha \;\left(x_{8}x_{13}\right)}{Z_{2}}$	$\frac{\alpha \;\left(x_{8}x_{14}\right)}{Z_{2}}$	$\frac{\;\left(x_{8}x_{15}\right)}{Z_{2}}$	$\frac{\;\left(x_{8}x_{16}\right)}{Z_{2}}$
	x_9_	$\frac{\alpha \;\left(x_{9}x_{2}\right)}{Z_{3}}$	$\frac{\;\left(x_{9}x_{2}\right)}{Z_{3}}$	$\frac{\alpha \;\left(x_{9}x_{3}\right)}{Z_{3}}$	$\frac{\;\left(x_{9}x_{4}\right)}{Z_{3}}$	$\frac{\alpha \;\left(x_{9}x_{5}\right)}{Z_{3}}$	$\frac{\;\left(x_{9}x_{6}\right)}{Z_{3}}$	$\frac{\alpha \left(x_{9}x_{7}\right)}{Z_{3}}$	$\frac{\;\left(x_{9}x_{8}\right)}{Z_{3}}$	$\frac{\alpha \left(x_{9}x_{9}\right)}{Z_{3}}$	$\frac{\left(x_{9}x_{10}\right)}{Z_{3}}$	$\frac{\;\alpha \left(x_{9}x_{11}\right)}{Z_{3}}$	$\frac{\;\left(x_{9}x_{12}\right)}{Z_{3}}$	$\frac{\alpha \;\left(x_{9}x_{13}\right)}{Z_{3}}$	$\frac{\;\left(x_{9}x_{14}\right)}{Z_{3}}$	$\frac{\;\alpha \left(x_{9}x_{15}\right)}{Z_{3}}$	$\frac{\;\left(x_{9}x_{16}\right)}{Z_{3}}$
	x_10_	$\frac{\alpha \;\left(x_{10}x_{2}\right)}{Z_{3}}$	$\frac{\;\left(x_{10}x_{2}\right)}{Z_{3}}$	$\frac{\alpha \;\left(x_{10}x_{3}\right)}{Z_{3}}$	$\frac{\;\left(x_{10}x_{4}\right)}{Z_{3}}$	$\frac{\alpha \;\left(x_{10}x_{5}\right)}{Z_{3}}$	$\frac{\;\left(x_{10}x_{6}\right)}{Z_{3}}$	$\frac{\alpha \left(x_{10}x_{7}\right)}{Z_{3}}$	$\frac{\;\left(x_{10}x_{8}\right)}{Z_{3}}$	$\frac{\alpha \left(x_{10}x_{9}\right)}{Z_{3}}$	$\frac{\left(x_{10}x_{10}\right)}{Z_{3}}$	$\frac{\;\alpha \left(x_{10}x_{11}\right)}{Z_{3}}$	$\frac{\;\left(x_{10}x_{12}\right)}{Z_{3}}$	$\frac{\alpha \;\left(x_{10}x_{13}\right)}{Z_{3}}$	$\frac{\;\left(x_{10}x_{14}\right)}{Z_{3}}$	$\frac{\;\alpha \left(x_{10}x_{15}\right)}{Z_{3}}$	$\frac{\;\left(x_{10}x_{16}\right)}{Z_{3}}$
	x_11_	$\frac{\alpha \;\left(x_{11}x_{2}\right)}{Z_{3}}$	$\frac{\;\left(x_{11}x_{2}\right)}{Z_{3}}$	$\frac{\alpha \;\left(x_{11}x_{3}\right)}{Z_{3}}$	$\frac{\;\left(x_{11}x_{4}\right)}{Z_{3}}$	$\frac{\alpha \;\left(x_{11}x_{5}\right)}{Z_{3}}$	$\frac{\;\left(x_{11}x_{6}\right)}{Z_{3}}$	$\frac{\alpha \left(x_{11}x_{7}\right)}{Z_{3}}$	$\frac{\;\left(x_{11}x_{8}\right)}{Z_{3}}$	$\frac{\alpha \left(x_{11}x_{9}\right)}{Z_{3}}$	$\frac{\left(x_{11}x_{10}\right)}{Z_{3}}$	$\frac{\;\alpha \left(x_{11}x_{11}\right)}{Z_{3}}$	$\frac{\;\left(x_{11}x_{12}\right)}{Z_{3}}$	$\frac{\alpha \;\left(x_{11}x_{13}\right)}{Z_{3}}$	$\frac{\;\left(x_{11}x_{14}\right)}{Z_{3}}$	$\frac{\;\alpha \left(x_{11}x_{15}\right)}{Z_{3}}$	$\frac{\;\left(x_{11}x_{16}\right)}{Z_{3}}$
	x_12_	$\frac{\alpha \;\left(x_{12}x_{2}\right)}{Z_{3}}$	$\frac{\;\left(x_{12}x_{2}\right)}{Z_{3}}$	$\frac{\alpha \;\left(x_{12}x_{3}\right)}{Z_{3}}$	$\frac{\;\left(x_{12}x_{4}\right)}{Z_{3}}$	$\frac{\alpha \;\left(x_{12}x_{5}\right)}{Z_{3}}$	$\frac{\;\left(x_{12}x_{6}\right)}{Z_{3}}$	$\frac{\alpha \left(x_{12}x_{7}\right)}{Z_{3}}$	$\frac{\;\left(x_{12}x_{8}\right)}{Z_{3}}$	$\frac{\alpha \left(x_{12}x_{9}\right)}{Z_{3}}$	$\frac{\left(x_{12}x_{10}\right)}{Z_{3}}$	$\frac{\;\alpha \left(x_{12}x_{11}\right)}{Z_{3}}$	$\frac{\;\left(x_{12}x_{12}\right)}{Z_{3}}$	$\frac{\alpha \;\left(x_{12}x_{13}\right)}{Z_{3}}$	$\frac{\;\left(x_{12}x_{14}\right)}{Z_{3}}$	$\frac{\;\alpha \left(x_{12}x_{15}\right)}{Z_{3}}$	$\frac{\;\left(x_{12}x_{16}\right)}{Z_{3}}$
	x_13_	(x_13_x_1_)	(x_13_x_2_	(x_13_x_3_)	(x_13_x_4_)	(x_13_x_5_)	(x_13_x_6_)	(x_13_x_7_)	(x_13_x_8_)	(x_13_x_9_)	(x_13_x_10_)	(x_13_x_11_)	(x_13_x_12_)	(x_13_x_13_)	(x_13_x_14_)	(x_13_x_15_)	(x_13_x_16_)
	x_14_	(x_14_x_1_)	(x_14_x_2_)	(x_14_x_3_)	(x_14_x_4_)	(x_14_x_5_)	(x_14_x_6_)	(x_14_x_7_)	(x_14_x_8_)	(x_14_x_9_)	(x_14_x_10_)	(x_14_x_11_)	(x_14_x_12_)	(x_14_x_13_)	(x_14_x_14_)	(x_14_x_15_)	(x_14_x_16_)
	x_15_	(x_15_x_1_)	(x_15_x_2_)	(x_15_x_3_)	(x_15_x_4_)	(x_15_x_5_)	(x_15_x_6_)	(x_15_x_7_)	(x_15_x_8_)	(x_15_x_9_)	(x_15_x_10_)	(x_15_x_11_)	(x_15_x_12_)	(x_15_x_13_)	(x_15_x_14_)	(x_15_x_15_)	(x_15_x_16_)
	x_16_	(x_16_x_1_)	(x_16_x_2_)	(x_16_x_3_)	(x_16_x_4_)	(x_16_x_5_)	(x_16_x_6_)	(x_16_x_7_)	(x_16_x_8_)	(x_16_x_9_)	(x_16_x_10_)	(x_16_x_11_)	(x_16_x_12_)	(x_16_x_13_)	(x_16_x_14_)	(x_16_x_15_)	(x_16_x_16_)
	Z_1_ = αp(x_1_ + x_5_ + x_9_ + x_13_) + α(x_2_ + x_3_ + x_6_ + x_7_ + x_10_ + x_11_ + x_14_ + x_15_) + x_4_ + x_8_ + x_12_ + x_16_																
	*Z*_2_ = *α*(*x*_1_ + *x*_2_ + *x*_5_ + *x*_6_ + *x*_9_ + *x*_10_ + *x*_13_ + *x*_14_) + *x*_3_ + *x*_4_ + *x*_7_ + *x*_8_ + *x*_11_ + *x*_12_ + *x*_15_ + *x*_16_																
	*Z*_3_ = *α*(*x*_1_ + *x*_3_ + *x*_5_ + *x*_7_ + *x*_9_ + *x*_11_ + *x*_13_ + *x*_15_) + *x*_2_ + *x*_4_ + *x*_6_ + *x*_8_ + *x*_10_ + *x*_12_ + *x*_14_ + *x*_16_																

I first confirmed that multiple preferences evolved in the face of costly competition. Multiple preferences evolve but require stronger preference strengths (i.e. greater *α*) to reach fixation than preferences evolving in the absence of costly competition (Figure 1, gray line). Interestingly, the strength of preference necessary to overcome the costs of choice is lower when multiple preferences are present versus a single preference. With simulations alone, however, it is impossible to determine if this is due to a decrease in competitive costs or an increase in indirect selection driven by stronger joint preferences by females with both preferences for males with both traits.

To distinguish between a decrease in competitive costs and an increase in indirect selection, I again calculated the strength of direct selection (Appendix 2). As before, selection is a function of trait and preference frequencies. Because of the number of loci, the solution for *a*_*P*,0_ is a complicated expression. For analytical tractability, I performed a weak selection approximation assuming weak preferences and small values for linkage disequilibrium:

(8) $$\begin{array}{c} a_{P_{1},0}\approx \frac{1}{{\left(T_{1}-1\right)}^{2}{\left(T_{2}-1\right)}^{2}}\cdot \gamma \\ \;\left(-P_{2}\left(1-T_{1}(3+2T_{2}\right))\left(P_{2}-T_{2}^{2}\right)\right)\\ \;+P_{1}\left(1-T_{1}\left(T_{1}+3\right)\right)\left(T_{2}\left(T_{2}-1\right)\right)\\ \;+P_{2}\left(3T_{2}\left(1-T_{2}\right)-1+P_{2}\left(1-3T_{2}(T_{2}-1\right)\right)) \end{array}$$

Again, as expected, selection on preference is a function of trait frequency. Without cost, or when both traits are fixed, selection on preference is 0. Under all other conditions, as before, selection is negative. Because of the complexity of (8), proving that it is always negative is not feasible; I used numerical simulations to verify that with two preferences and traits, *a*_*P*1,0_≤0.

**Figure 3:**
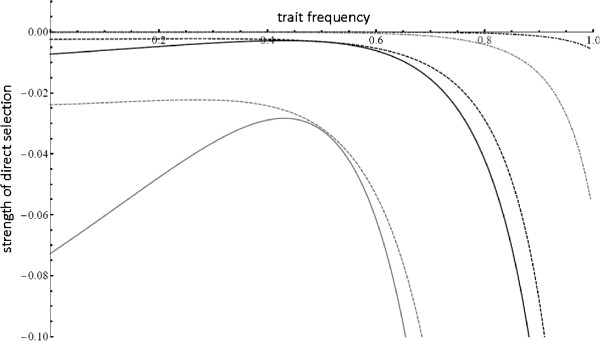
**Direct selection for a single preference; two preference, two trait model.** This figure shows the strength of direct selection against a single female preference (*a*_*p*1,0_) as male trait frequency increases. Each line represents selection against P1 for a different combination of preference frequencies (P1 and P2) and cost (γ) as male trait frequencies (T1 and T2) increase. The solid black line indicates P1=.95, P2=.01, γ=.01; solid gray indicates P1=.95, P2=.01, γ=.1; dashed black indicates P1=.01, P2=.01, γ=.01; dashed grey indicates P1=.01, P2=.01, γ=.1; dot-dashed black indicates P1=.95, P2=.95, γ=.01; and dot-dashed grey indicates P1=.95, P2=.95, γ=.1. For all simulations, *α*=5; changing preference strength did not change the shape of the cost curve, but only scaled up the strength of selection against preference. Regardless of the parameters, direct selection on P1 is always negative, except when male traits are absent. All the cost curves turn downwards as male trait frequency increases—this is due to selection for multiple preferences (P1P2 together) when male trait frequencies are sufficiently high; the curves displayed are for a single preference (P1).

To visualize the strength of selection, I plotted the original (not weak selection) equation for direct selection for different preference and trait frequencies, and competitive costs (Figure 3). Just as in the weak selection approximation, all values of *a*_*P*,0_ are negative, except in the case of γ=0 or *α*=1, in which case *a*_*P*,0_=0.

Comparing figures two and three, it is clear that the presence of a second preference alters the strength of direct selection, but does not lead to direct selection for multiple preferences. In general, it appears that the presence of a second preference does decrease costs, but only when preferences are common. When preferences are rare, the presence of a second preference can increase competitive costs drastically by leading to female with two preferences having very strong preferences for rare two-trait males; this in turn would lead to fierce competition. Thus, a second preference would not directly reduce competitive costs when introduced at a low frequency. When preferences are already at a high frequency, there is a benefit to having multiple preferences (see Figure 3), but here I focused on low initial frequencies as an evolutionarily realistic scenario.

### Indirect selection

**Figure 4:**
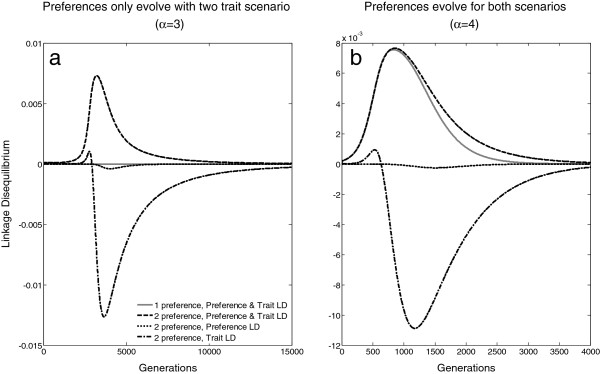
**Linkage disequilibria between preference and trait alleles.** This figure displays the linkage disequilibriabetween preference and trait alleles in the single preference/trait (two locus) and two preference/trait(four locus) models. 4**a** shows linkage disequilibria where the cost of competition=.1, and *α*=3; under these conditions preferences only fix when two are evolving simultaneously. 4**b** shows linkage disequilibria where the cost of competition=.1, and *α*=5; in this case, both a single preference/trait pair and two preference/trait pairs can evolve to fixation. In both scenarios, the linkage disequilibrium between preference and trait is greater in the 2 preference model (black dashed line) than the 1 preference model (solid gray line).

A major force behind the increased costs associated with multiple preferences is likely to be linkage disequilibrium (LD) between female preferences and male traits. When a male has both trait alleles, he is attractive to females with either one or both preference alleles, and in turn produces offspring who have both male trait alleles along with the preference alleles, which in time creates a non random association between multiple male traits and female preferences. Figure 4 shows this process over time. When preferences evolve to fixation, LD forms between preference and trait loci as they are spreading through the population. When there are two preference/trait pairs evolving simultaneously, LD is also created between the two traits, due to strong female preferences for the two trait males. As male traits become more common, they end up in negative LD (more likely to have only a single trait) due to competition reducing the number of two-preference females. When I compared LD between preference and trait when only a single pair was evolving, vs two pairs evolving simultaneously, I found that it is always greater when two are evolving simultaneously. This increased indirect selection can then allow preferences to evolve when multiple preferences are present even though costs do prevent their evolution when only a single preference is present. Despite strong direct selection against preference evolution, the LD created by multiple preferences may increase indirect selection enough to overcome natural selection and allow preferences to persist despite costs.

### Simulation studies

To make realistic predictions about the evolution of multiple preferences I used simulation models to explore when multiple preferences could evolve. Using the model framework described above, I looked at the evolution of preferences with female competition. To explore the full range of possibilities for preference and trait evolution, I considered 3 scenarios:

1 female preference for arbitrary male traits,

2 female preference for male traits favored by natural selection,

3 female preference for condition dependent traits, and

For each scenario, I simulated the evolution of two preferences introduced simultaneously to the evolution of two preferences introduced successively (i.e. the second preference is only introduced after the first one is at equilibrium). I performed numerical simulations in Matlab; equilibrium conditions were found by running recursion equations for genotype frequency, as described above, until trait and preference alleles reached equilibrium. The results presented below are derived from genotype frequencies at equilibrium, which I defined as when the percentage change in genotype frequencies between successive generations was less than 10^-16^.

#### Female preferences for arbitrary male traits

**Figure 5:**
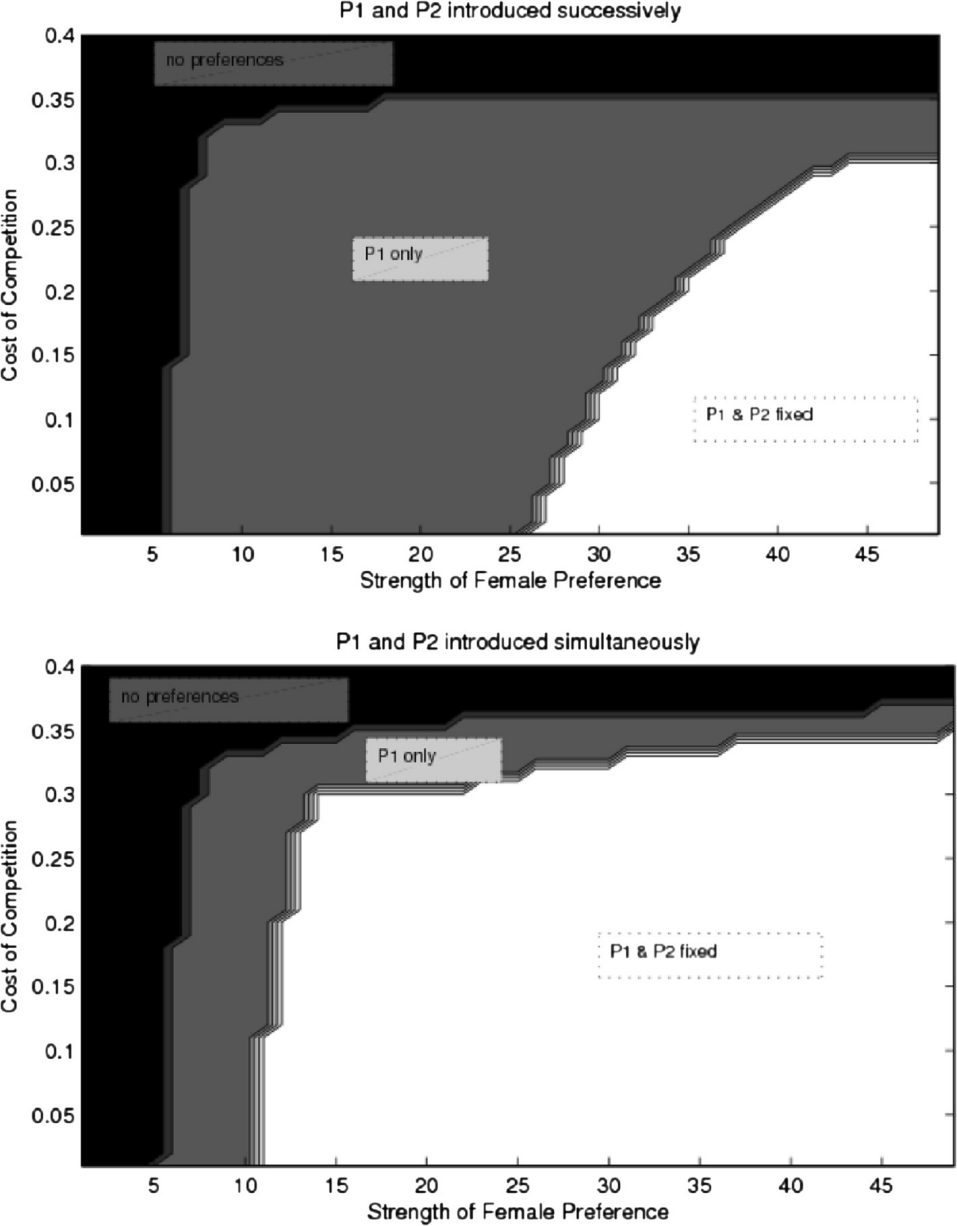
**Simulation results for the evolution of multiple preferences for arbitrary male traits.** This figure shows the parameter space, in terms of costs of competition (γ) and strength of female preference (*α*), where a single preference for an arbitrary trait fixes (gray), both preferences fix (white), and both preferences are lost (black). Top panel is for successive preference introduction, bottom panel shows simultaneous preference introduction. For a preference to fix, preference strength must be sufficiently high, and costs must be relatively low. Introducing preferences successively increases the parameter space where multiple preferences may coexist.

I began by simulating a four locus model of female preferences for arbitrary male traits, as described and modeled analytically above. Females gained nothing from mating with preferred males other than producing attractive offspring, and there was no natural selection. With successive introduction of female preference, the initial female preference evolved to fixation when preference was high enough, despite the cost of competition. When a second preference was introduced, after the fixation of the first, even higher preference strength was needed to overcome competitive costs; competition for the limited pool of males with both traits prevented preference evolution unless preferences for male traits were very strong (Figure 5a). When introduced simultaneously, both preferences could fix when the strength of female preference was high and the cost of competition relatively low (a>5,γ<0.35, see Figure 5b). As predicted in Figure 1, with two preferences introduced simultaneously, the minimum preference strength for preference evolution decreased somewhat. It is also worth noting that the two preferences were able to fix under a broader set of circumstances when introduced simultaneously vs. successively – this replicates the result in Figure 2, where direct selection against preference is weaker when two preferences are at low frequencies (dashed lines) than when one is already at a high frequency (solid lines).

I also considered the role of the cost function itself (as defined in equation 2) in determining the conditions under which preference may evolve. Numerically, I simulated a convex cost function and a concave cost function, and compared the parameters under which preferences could evolve. As one might expect, a concave cost function expanded the parameter space where preferences evolved while a convex function further restricted the space where preferences could evolve. Regardless of the shape of the cost function, as long as fertility was reduced in some way due to competition, the parameter space where preferences could evolve was restricted.

#### Female preferences for male traits favored by natural selection

To model honest traits, I first considered male traits favored by natural selection. I altered the four locus model such that following birth, individuals underwent natural selection: individuals of type *i* without traits had fitness reduced by a fraction *s*_*i*_. Genotype frequency following natural selection was then described by:

(9) $$x_{i}^{\eta }=\frac{\left(1-s_{i}\right)x_{i}}{\sum_{i=1}^{16}\left(1-s_{i}\right)x_{i}}\text{.}$$

Where i∈4,8,12,16. The *x*_*i*_^*η*^ values in (4) replace the *x*_*i*_ values in (1).

**Figure 6:**
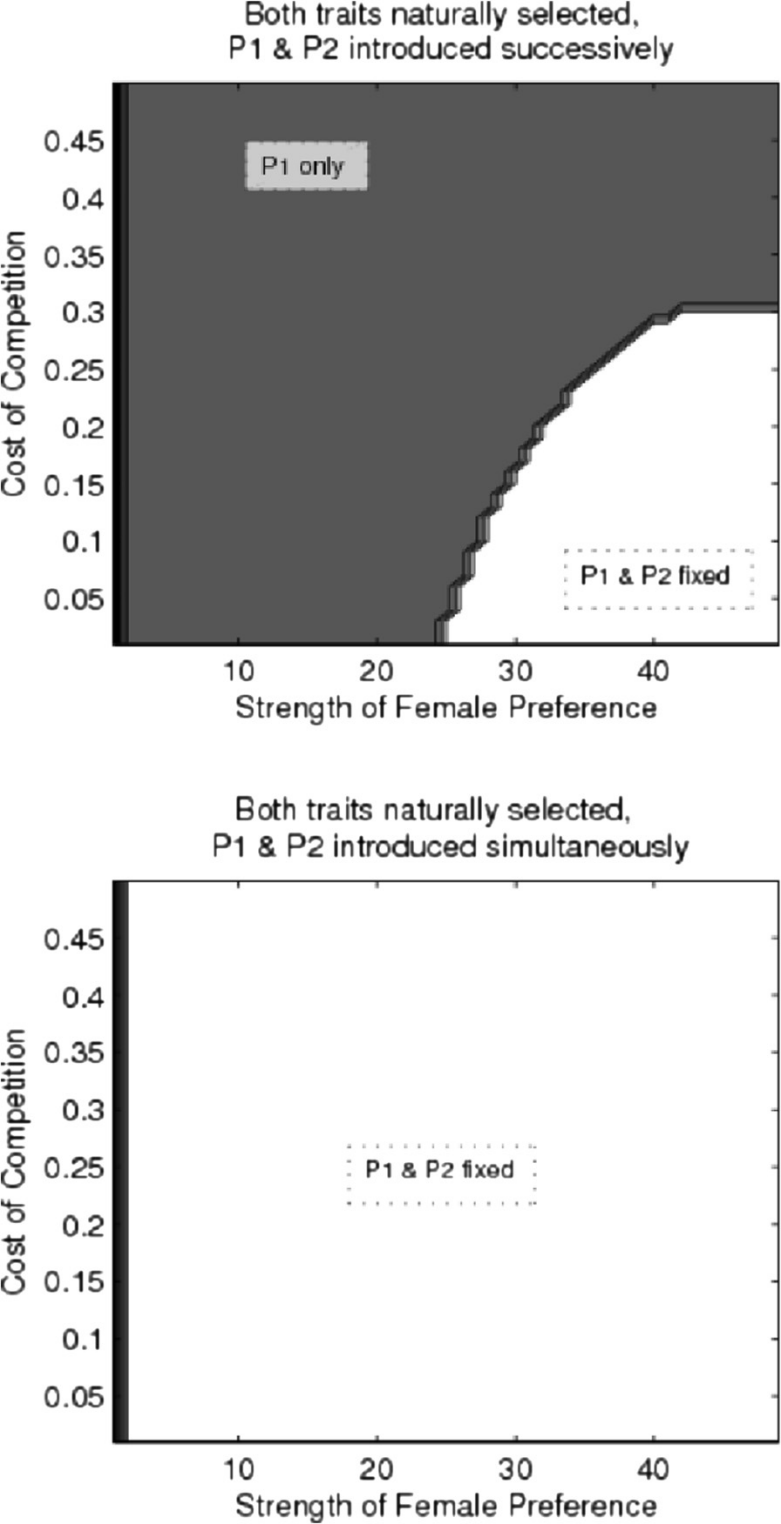
**Evolution of multiple preferences for naturally selected male traits.** This figure shows the parameter space, in terms of costs of competition (γ) and strength of female preference (*α*), where a single preference for a naturally selected trait fixes (gray), both preferences fix (white), and both preferences are lost (black, on the far left along the y-axis). The top figure displays results for successive preference introduction, the bottom shows simultaneous preference introduction. Natural selection on traits counterbalances direct selection against preferences due to competition, allowing at least a single preference to fix under most parameter combinations.

The direct benefits of a male favored by natural selection is sufficient to overcome direct selection against preferences due to female competition—a single preference for naturally selected male traits fixed across a wide range of parameter combinations. Figure 6 displays only a∈[0,50], and γ∈[0,0.5], but a much wider range was examined for both parameters, and unless γ was unrealistically high (e.g., γ>1.5), a single preference was able to fix. However, direct selection against preferences was able to prevent the evolution of a second preference when the first was fixed—as in Figure 5, two preferences were able to fix more readily when introduced simultaneously.

#### Condition dependent male traits

For this scenario, I added a fifth locus C, which denotes an individual’s condition. Individuals with c are considered low condition; those with C are high condition, and thus favored by natural selection. The result is 16⋅2=32 genotypes. I included directional mutation from C to c in order to maintain variation in condition.

The life cycle consists of birth, natural selection, mate choice, fertility selection, zygote formation, recombination and mutation. During natural selection, low quality individuals (those with the c allele), were (1–*s*) times as likely to survive. For mate choice, males displayed traits only if they were also in good condition, ie, females did not prefer low condition males, even if they carried trait genes. Mate choice occurs as described in (1), using *k*_*ij*_ values given in Table 3. After mate choice, fertility selection occurs as in (3) and (4), followed by recombination, mutation, and zygote formation.

**Table 3:** **Values for k**_**ij**_**for condition dependent mate choice; i represents the female preference genotype, j represents male trait genotype**

**Condition dependent mate choice**				
	**T**_**1**_**T**_**2**_**C (x**_**1**_**, x**_**9**_**,x**_**17**_**,x**_**25**_**)**	**T**_**1**_**t**_**2**_**C (x**_**3**_**, x**_**11**_**,x**_**19**_**,x**_**27**_**)**	**t**_**1**_**T**_**2**_**C (x**_**5**_**, x**_**13**_**,x**_**21**_**,x**_**29**_**)**	**t**_**1**_**t**_**2**_**C(x**_**7**_**, x**_**15**_**,x**_**23**_**,x**_**31**_**), all c genotypes(*x***_**2**_**,x**_**4**_**,x**_**6**_**, . . .,x**_**32**_**)**
P_1_P_2_ (x_1_, *x*_2_,x_3_,x_4_, x_5_, x_6_,x_7_,x_8_)	1.5	1	1	0
P_1_p_2_ (x_9_, x_10_,x_11_,x_12_, x_13_, x_14_,x_15,_x_16_)	1	1	0	0
p_1_P_2_ (x_17_, x_18_,x_19_,x_20_, x_21_, x_22_,x_23_,x_24_)	1	0	1	0
p_1_p_2_ (x_25_, x_26_,x_27_,x_28_, x_29_, x_30_,x_31_,x_32_)	0	0	0	0

Because condition-dependent trait expression leads to increased mate competition because there is a decreased pool of males expressing traits, I considered two regimes for the evolution of condition-dependent preferences:

1 evolution of preference along with condition, where preference and condition are introduced at low frequency simultaneously and allowed to evolve together, and

2 evolution of preference in a system where the condition allele is at mutation selection balance (mutation rate for c is 0.005).

By examining both the evolution of condition allele with preference, and the introduction of preference into a high condition population, I can better distinguish the interaction between multiple preferences and condition evolution.

**Figure 7:**
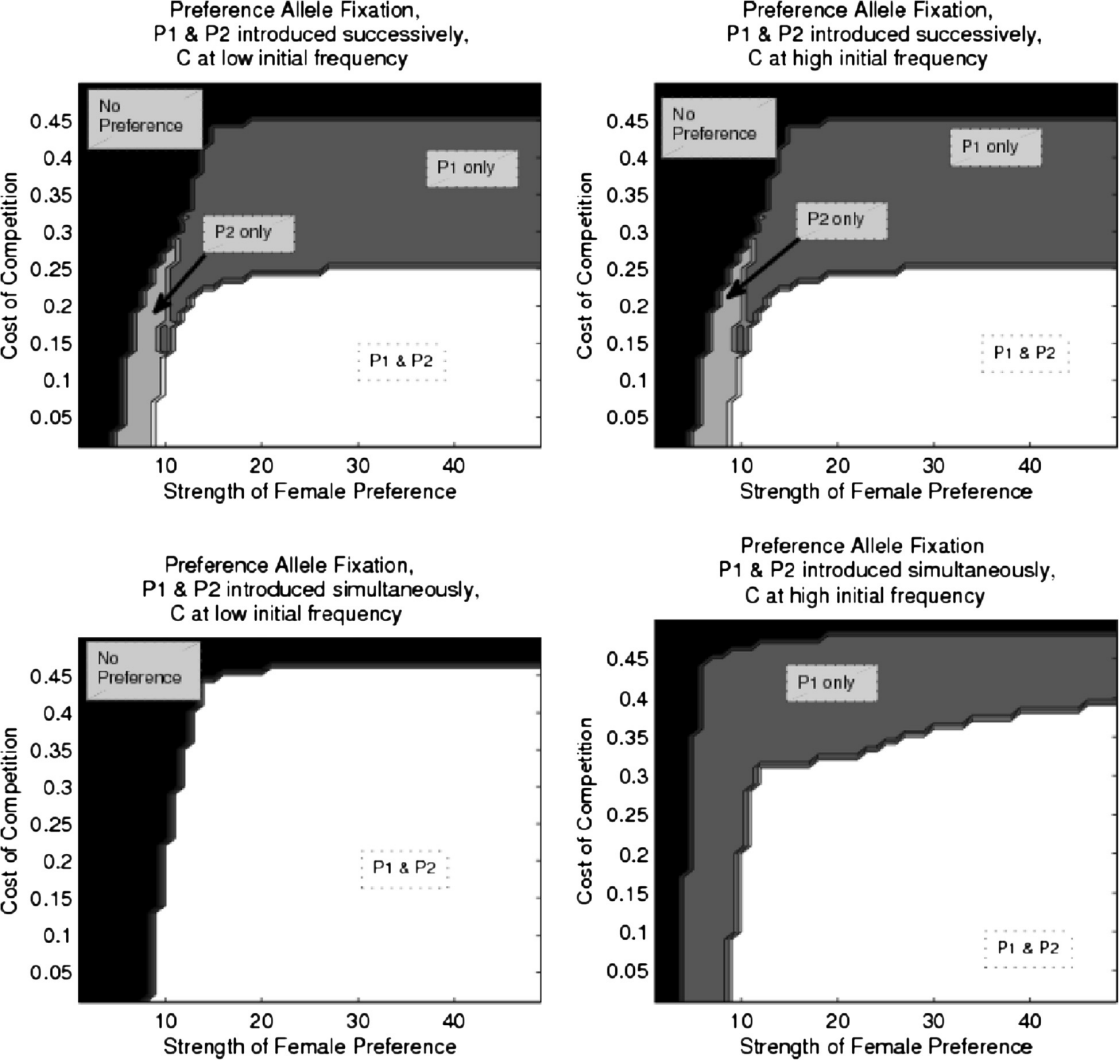
**Evolution of multiple preferences for condition dependent male traits.** This figure shows the parameter space, in terms of costs of competition (γ) and strength of female preference (*α*), where a single preference for a condition dependent trait fixes (gray), both preferences fix (white), and both preferences are lost (black). Top row displays results for successive preference introduction, the bottom row shows simultaneous preference introduction. The left column displays results for simulations where the condition allele evolved along with female preference and the right column shows results from simulations where female preference was introduced into a population at mutation selection balance for a high condition allele.

For both regimes, having two preferences evolving simultaneously (as opposed to successively) increased the size of the parameter space where a second preference fixed, allowing it to fix at higher levels of competitive costs (Figure 7). Further, as with naturally selected traits, direct selection for preferences for condition dependent male traits balances out direct selection against female preference due to competition, allowing preference to evolve under lower strengths and higher competition.

## Discussion

The results from my models indicate that intrasexual competition is costly and, when present, direct selection acts against preference evolution. Multiple preferences change the shape of the cost curve but fail to alleviate costly competition when introduced at a low frequency; direct selection still acts against female preference when multiple preferences are present. This is not to say that intrasexual competition entirely prevents preference evolution; simulation results indicated that preferences may still evolve if they are sufficiently strong enough to overcome natural selection, and that the multiple preferences evolving simultaneously may reduce (but not eliminate) direct selection. Although multiple preferences do not lead to direct (i.e. natural) selection for preference evolution, their presence is likely to increase the strength of indirect selection on preference and trait evolution, creating strong joint preferences in females with both preferences for males with both traits; this leads to a decrease in the initial preference strength required for evolution.

In general, these results are consistent with other models, where costs associated with mate choice have been shown to prevent or restrict the evolution of multiple female preferences (Kirkpatrick, 1985); [24,25]. Kirkpatrick’s (1985) model of the sexy son hypothesis showed that handicap traits, which only lower fitness, do not spread. Models explicitly considering multiple male traits with costly female preference, in terms of search costs/viability selection, also found that female preferences did not evolve due to high joint costs to preference [24,25]. In these models, if it was more costly for a female to search for and find a mate with multiple preferred traits rather than a male with a single trait, then multiple preferences could not evolve. Similarly, in my model, having multiple preferences served to increase competitive costs when male traits were rare.

My model supports the idea that intrasexual competition is likely to be a significant cost acting against the evolution of female preferences. There are many examples of intrasexual competition: direct aggression between females [7,8], reduced fecundity due to decreased male parental efforts (Summers 1990), [5], as well as decreased fecundity from male sperm depletion [11], (Royer and McNeil 1997), [9,12-14]. Yet, in the majority of these species, female preferences have evolved regardless – including multiple preferences. In my models, competitive costs are not an insurmountable obstacle; although multiple preferences fail to alleviate competition, they don’t appear to be significantly more costly than a single preference, and in fact serve to increase indirect selection on preferences (see Figure 1; the minimum *α* required for preference evolution is lower for multiple preferences).

Multiple preferences may in fact serve to alleviate competition, just not in the way modeled here. One possibility is that if individual females have different preferences, controlled by a single locus, instead of multiple preferences controlled by multiple loci, competition could be averted. However, this scenario is unlikely: in most species with multiple preferences, these preferences appear to be controlled by independent genes Brooks and Coulridge, [18,19]. As my model has shown, if preferences are controlled by independent loci, after several generations, many individuals have both preferences leading to increased competition, not avoidance.

Perhaps multiple preferences may not indirectly prevent competition, but instead involve preferences for traits which indicate how many times a male has mated. One study showed that female cockroaches discriminated against males that had mated multiple times, and were able to detect cues on males derived from previous mates, in addition to traits indicating male quality [12]. However, it is difficult to imagine how common the ability to detect prior matings is, and there is only one such example in the literature. Another possibility is that females could evolve multiple preferences and switch between preferences when they sense competition for a desired male. This would require knowledge about population wide preference frequencies, but would be possible in lekking species or animals that live in social groups.

## Conclusions

When multiple preferences are present, indirect selection on female preference evolution is much stronger. Perhaps instead of relieving competition, multiple preferences allow female choice to evolve by jointly increasing the strength of indirect selection to the point where many weak preferences can overcome natural selection against competition.

## Appendix

Appendix 1. a_p,0_ equations for a single preference and trait, two locus model

The relative fitness of female preference and male traits is:

(A1) $$\begin{array}{c} W\left(X_{p},X_{t^{*}}\right)=\frac{\left(1-X_{p}\right)\left(1-X_{t^{*}}\right)\;\phi_{1}}{Z_{1}}\\ \;+\frac{\left(1-X_{p}\right)X_{t^{*}}\;\phi_{2}}{Z_{1}}\\ \;+\frac{X_{p}\left(1-X_{t^{*}}\right)\;\phi_{3}}{Z_{2}}+\frac{X_{p}X_{t^{*}}\phi_{4}}{Z_{2}} \end{array}$$

where *X*_*p*_ represents the presence of preference alleles in females; *X*_*p*_=1 if a female has allele P, and 0 if she does not. Likewise, *X*_t*_=1 if a male has allele T, and 0 if he does not. *z*_*i*_ is the normalization for sexual selection (as described in equation 2). *ϕ*_*i*_ is the fertility selection against male genotype i (see equation 3 in the text). For example, for an x_1_ individual (PT), *X*_*p*_=1 and *X*_t*_=1, and $X_{p},X_{t^{*}}=\frac{\phi_{4}}{Z_{2}}\text{.}$

Equation (A1) can be used to calculate the a terms present in equation (4) in the text. To calculate the as, the fitness equation for a model (here, A1) is set equal to a generic equation for fitness in terms of as and Cs, and a function of the Xs. Terms are then matched to solve for a in the model under consideration. This procedure is described fully in appendix B of Kirkpatrick and Servedio.

Appendix 2. a_p,0_ equations for a single preference and trait, four locus model

The relative fitness of a female possessing a preference allele in the four locus model is:

(A2) $$\begin{array}{c} W\left(X_{P_{1}},X_{P_{2}},X_{T_{1}},X_{T_{2}}\;\right)=\frac{\left(1-X_{{\text{P}}_{1}}\right)\left(1-X_{P_{2}}\right)\left(1-X_{T_{1}^{*}}\right)\left(1-X_{T_{2}^{*}}\right)\;p\;\alpha \;\phi_{1}}{Z_{1}}\\ \;+\frac{\left(1-X_{{\text{P}}_{1}}\right)\left(1-X_{P_{2}}\right)\left(1-X_{T_{1}^{*}}\right)X_{T_{2}}^{*}\alpha \;\phi_{2}}{Z_{1}}\\ \;+\frac{\left(1-X_{{\text{P}}_{1}}\right)\left(1-X_{P_{2}}\right)X_{T_{1}^{*}}\left(1-X_{T_{2}^{*}}\right)\;\alpha \;\phi_{3}}{Z_{1}}\\ \;+\frac{\left(1-X_{{\text{P}}_{1}}\right)\left(1-X_{P_{2}}\right)X_{T_{1}^{*}}X_{T_{2}^{*}}\;\phi_{4}}{Z_{1}}\\ \;+\frac{\left(1-X_{{\text{P}}_{1}}\right)X_{P_{2}}\left(1-X_{T_{1}^{*}}\right)\left(1-X_{T_{2}^{*}}\right)\;\alpha \;\phi_{1}}{Z_{2}}\\ \;+\frac{\left(1-X_{{\text{P}}_{1}}\right)X_{P_{2}}\left(1-X_{T_{1}^{*}}\right)\;X_{T_{2}^{*}}\;\alpha \;\phi_{2}}{Z_{2}}\\ \;+\frac{\left(1-X_{{\text{P}}_{1}}\right)X_{P_{2}}X_{T_{1}^{*}}\left(1-X_{T_{2}^{*}}\right)\;\phi_{3}}{Z_{2}}\\ \;+\frac{\left(1-X_{{\text{P}}_{1}}\right)X_{P_{2}}X_{T_{1}^{*}}X_{T_{2}^{*}}\;\phi_{4}}{Z_{2}}\\ \;+\frac{X_{{\text{P}}_{1}}\left(1-X_{P_{2}}\right)\left(1-X_{T_{1}^{*}}\right)\left(1-X_{T_{2}^{*}}\right)\;\alpha \;\phi_{1}}{Z_{3}}\\ \;+\frac{X_{{\text{P}}_{1}}\left(1-X_{P_{2}}\right)\left(1-X_{T_{1}^{*}}\right)X_{T_{2}}^{*}\;\phi_{2}}{Z_{3}}\\ \;+\frac{X_{{\text{P}}_{1}}\left(1-X_{P_{2}}\right)X_{T_{1}^{*}}\left(1-X_{T_{2}^{*}}\right)\;\alpha \;\phi_{3}}{Z_{3}}\\ \;+\frac{X_{{\text{P}}_{1}}\left(1-X_{P_{2}}\right)X_{T_{1}^{*}}X_{T_{2}^{*}}\;\phi_{4}}{Z_{3}}\\ \;+\frac{X_{{\text{P}}_{1}}X_{P_{2}}\left(1-X_{T_{1}^{*}}\right)\left(1-X_{T_{2}^{*}}\right)\;\phi_{1}}{z_{4}}\\ \;+\frac{X_{{\text{P}}_{1}}X_{P_{2}}\left(1-X_{T_{1}^{*}}\right)X_{T_{2}}^{*}\;\phi_{2}}{z_{4}}\\ \;+\frac{X_{{\text{P}}_{1}}X_{P_{2}}X_{T_{1}^{*}}\left(1-X_{T_{2}^{*}}\right)\;\phi_{3}}{z_{4}}\\ \;+\frac{X_{{\text{P}}_{1}}X_{P_{2}}X_{T_{1}^{*}}X_{T_{2}^{*}}\;\phi_{4}}{z_{4}} \end{array}$$

As in Appendix 1, *X*_*Pi*_ represents the presence of preference alleles in females, where *X*_*P1*_=0 if a female has preference i, and 0 if she does not. Likewise, *X*_*Ti*_=0 if a male has trait i, and 0 if he does not. *Z*_*i*_ is the normalization for sexual selection (Z_1_, Z_2_, and Z_3_ are described in Table 2; Z_4_=1). *ϕ*_*i*_ is the fertility selection against male genotype i (see equation 3 in the text). As with female preference, there are only four unique male genotype combinations such that *ϕ*_*1*_ is the discount for T1T2 males, *ϕ*_*2*_ is for T1t2 males, *ϕ*_*3*_ is for t_1_T_2_ males, and *ϕ*_*4*_ is the discount for t_1_t_2_ males.

As in Appendix 1, equation (A2) is used to calculate the a terms present in equation (5) in the text. Because of the complexity of equation (A2), I applied a weak selection approximation to get a shorter, analytically tractable expression for *a*_*P*,0_: I assumed that costs were low, preferences weak, and linkage disequilibrium small (confirmed via simulations), and performed a taylor series expansion of *a*_*P*,0_. This method yielded equation (8), a considerably shorter expression for direct selection on preferences. To confirm the validity of the weak selection approximation, I compared it to the original expression and confirmed that, as α, γ, and D_i,j_ decreased, the two expressions converged. For the sake of comparison to (6), the equation used in Figure 3 is the original formulation of *a*_*P*,0_, not the weak selection approximation.

## Competing interests

The author declares that she has no competing interests.

## Author contributions

AMF conducted all of the research presented in this paper and wrote the manuscript. She has read and approved the final manuscript.
